# Lornoxicam suppresses recurrent herpetic stromal keratitis through down-regulation of nuclear factor-κB: an experimental study in mice

**Published:** 2009-06-14

**Authors:** Jie Yin, Zhenping Huang, Yuan Xia, Fei Ma, Li Jing Zhang, Heng Hui Ma, Li Li Wang

**Affiliations:** 1 Department of Ophthalmology, Jinling Hospital, School of Medicine, Nanjing University, Nanjing, People’s Republic of China; 2 Department of Pathology, Jinling Hospital, School of Medicine, Nanjing University, Nanjing, People’s Republic of China

## Abstract

**Purpose:**

We designed the current study to determine the protective effects of lornoxicam, a cyclooxygenase (COX) inhibitor, on recurrent herpetic stromal keratitis (HSK) and the nuclear factor-κB (NF-κB)-mediated mechanism in mice.

**Methods:**

A corneal latent herpes simplex virus-1 (HSV-1) infected mouse model was established. Six weeks later, Ultraviolet B (UVB) irradiation induced the recurrence. Corneal swabs were obtained and cultured with indicator cells to determine shedding of the virus. Lornoxicam was administered intraperitoneally daily, beginning one day before irradiation and lasting for seven days. Saline-treated and mock-infected control groups were also studied at the same time. Development of corneal inflammation and opacity was scored. Immunohistochemical staining and an electrophoretic mobility shift assay were performed to evaluate the effect of lornoxicam on NF-κB activation in the corneal tissues. The levels of tumor necrosis factor-α (TNF-α) in the cornea were determined by an enzyme-linked immunosorbent assay (ELISA).

**Results:**

HSV-1 reactivation induced stromal edema and opacification concomitantly with elevated activation of NF-κB and elevated production of TNF-α. Lornoxicam treatment significantly decreased the incidence of recurrent HSK, attenuated the corneal opacity scores, and also effectively suppressed both NF-κB activation and TNF-α expression in biological analysis. Histopathology examination revealed a reduced immunostaining positive cell density for NF-κB in the cornea from lornoxicam-treated mice as well as a diminished inflammatory response.

**Conclusions:**

Lornoxicam exerts protective effects against HSK, presumably through the down-regulation of NF-κB activation.

## Introduction

Herpes simplex virus (HSV)-1 is one of the leading causes of infectious corneal blindness. Recurrent corneal herpetic disease is more harmful than the primary infection [1]. Unrestrained inflammatory responses in recurrent herpetic stromal keratitis (HSK) may result in irreparable histological damage including corneal neovascularization, scarring, and permanent opacities that lead to blindness. Traditional antiviral therapy is limited because the pathogenesis of recurrent HSK involves mainly immune-mediated mechanisms rather than viral cytopathic effects [2]. Viral replication is an initial factor to induce recurrent HSK. Subsequently, activated inflammatory cells begin to orchestrate the immunopathological process even in the absence of a replicating virus in the developing period of the disease [3]. Therefore, regulating the excessive immune response is crucial in controlling progressive visual impairment in recurrent HSK [2]. Previous reports have shown antiviral activity of a cyclooxygenase (COX) inhibitor against ocular infection with HSV-1 [4-6]. COX inhibitors, which belong to anti-inflammatory drugs, may contribute to controlling the massive immune response at an effective yet not deleterious level. However, the concrete influence of a COX inhibitor on the inflammatory mediators and the mechanistic basis needs further investigation.

Nuclear factor-κB (NF-κB), a critical transcriptional regulatory factor, regulates the expression of diverse genes such as those involved in cell growth, inflammation, and immune reaction. These genes include cytokines such as tumor necrosis factor-α (*TNF-α*), inducible genes such as *COX-2* and nitric oxide synthase, and intercellular adhesion molecule such as E-selectin. It is well-known that the synthesis of robust amounts of proinflammatory mediators such as TNF-α, interleukin (IL)-1β, and chemokines is crucial in enhancing the excessive host immune response during recurrent HSK [7,8]. Therefore, NF-κB may be a particularly important intermediate in the immunopathology of HSK. NF-κB seems to play a key role in the signaling pathways activated by HSV-1, influencing host immune response. Previous reports have demonstrated that the persistent nuclear translocation of NF-κB induced by HSV-1 infection is essential for efficient viral replication [9-12]. Hence, NF-κB can be a rational target for antiviral therapy. Collectively, it is challenging to know if the potent anti-inflammatory effect of a COX inhibitor against viral infection acts through the down-regulation of NF-κB activity.

In the present work, we tested our hypothesis by using lornoxicam (LOR), a widely used COX inhibitor in a murine HSK model. LOR exhibits a profound anti-inflammatory effect and is a potent, balanced inhibitor of COX-1 and COX-2, which may mitigate the concerns associated with chronic specific inhibition of COX-2 [13]. Moreover, it has great appeal because of its improved gastrointestinal safety with less risk of systemic side effects compared to the other oxicams [14,15]. We hypothesized that LOR would exert therapeutic effects on recurrent HSK by altering TNF-α expression through the down-regulation of NF-κB activity. This study may facilitate identification of novel molecular targets for HSK therapy.

## Methods

### HSV-1 virus, animals, and corneal HSV-1 infection

The HSV-l strain McKrae (kindly provided by Dr. Lixin Xie, Shandong Eye Institute, Qingdao, China.) was grown and assayed in human embryo kidney (HEK)-293 cells in Dulbecco’s modified minimum essential medium (MEM) containing 10% fetal bovine serum, 100 U/ml of penicillin, and 100 mg/ml of streptomycin. Cells were cultured at 37 °C in a humidified incubator containing 5% CO_2_. To detect recurrent ocular virus shedding, materials from eye swabs were plated onto the HEK-293 cells, and cytopathic effects were then monitored for a seven-day incubation period.

Six-week-old, female ICR mice, weighing about 20 g each, were obtained from the Shanghai Experimental Animal Center (Shanghai, China). The mice were fed a regular diet and kept under standard conditions with a 12-h light/12-h dark cycle (light from 07:00−19:00). The animals’ quarters were maintained at 21 °C−24 °C with 40%−60% humidity. All experiment procedures conformed to the Association for Research in Vision and Ophthalmology (ARVO) Statement for the Use of Animals in Ophthalmic and Vision Research. Prior to the experiment, all mice underwent an examination with slit-lamp biomicroscopy (Model 900 BQ; Haag-Streit, Bern, Switzerland) and any mice with anomalies of the anterior segment of the eye (cornea, anterior chamber, iris, or lens) were excluded from the study.

Two-hundred mice (180 infected and 20 mock-infected animals) were included in the study. Animals were infected using the methods as previously described [16]. Briefly, after the mouse was intraperitoneally anesthetized with 0.5% pentobarbital (45 mg/kg bodyweight), the corneal surface of the right eye was incised in a cross-shaped pattern with a sterile 26 gauge needle. The medium (5 μl) containing 10^6^ plaque forming units (pfu) of the HSV-1 McKrae strain was pipetted directly onto the wounded cornea. Mock-infected control mice were inoculated similarly with the cell culture medium (5 μl) of uninfected HEK-293 cells. Each mouse in the infected group received an intraperitoneal injection of 1 ml of pooled human serum concurrent with infection to ensure its survival [17]. Mock-infected control mice received human serum as well.

### UVB irradiation and virus reactivation

Six weeks after inoculation, reactivation of latent HSV-1 infection was induced by Ultraviolet B (UVB) irradiation. The primary infection was confirmed by positive culture of HSV eye swabs obtained three days after inoculation. The eyes of all mice were then examined for corneal opacity before irradiation, and only animals with clear corneas lacking permanent damage were used. Finally, the eyes of 152 latently infected and 20 mock- infected mice were UV-irradiated and examined for signs of disease and viral reactivation in the reactivation phase. In brief, the right eye of every anesthetized mouse was exposed to 300 mJ/cm^2^ of UVB light (Model Waldmann UV−100L; Waldmann Co., Villingen-Schwenningen, Germany). The irradiation intensity at the corneal surface measured using a Waldmann UV detector device from the same company was 10.5 mW/cm^2^. The wavelength of this narrow-band phototherapy device ranges from 310 nm to 315 nm.

The eye swab materials from the mice were obtained before (day 0) and on days 1–7 post-UV irradiation and were cultured in HEK-293 cells as described above to detect recurrent virus shedding from the cornea. Recurrent disease was defined as stromal opacification for more than two consecutive days and virus shedding in tears on any day from day 1 to day 7 post-UVB exposure (day 0 swabs served as controls).

### Drug intervention and clinical observation of corneal disease progression

The infected and control mice were treated with LOR (0.4 mg/kg/day; Nycomed Austria GmbH, Linz, Austria) by intraperitoneal injection once every day from one day before irradiation to day 6 post-UVB irradiation. The optimal dose of LOR was determined in a preliminary experiment. In brief, 50 HSV-1-infected animals were randomly divided into five groups (10 per group), irradiated with UVB, and then treated with 0.04, 0.2, 0.4, 2.0, or 4.0 mg/kg of LOR. No toxic side effects or intolerance reactions occurred in any of the mice. Out of the 10 animals in each group, 7 (0.04 mg/kg and 0.2 mg/kg LOR groups), 4 (0.4 mg/kg and 2.0 mg/kg LOR groups), and 5 animals (4.0 mg/kg LOR group) presented with recurrent disease. Therefore, the dosage of 0.4 mg/kg appeared to be the minimal effective dose for preventing viral reactivation. The 0.4 mg/kg dosage was used in the subsequent experiment. Saline was used by intraperitoneal injection as a placebo in the control group.

In the reactivation phase of the study, the infected and control mice were randomly divided into the following four groups. The first group was the test group consisting of 76 mice that were latently infected with the virus and treated with LOR. The second group was the saline-treated group consisting of 76 mice that were infected with the virus and treated with saline. The third group was the model control group consisting of 10 mice that were mock infected and treated with saline. The final group was the LOR control group consisting of 10 mice mock infected and treated with LOR. The drug treatment was prophylactically started one day before UVB irradiation and repeated for seven consecutive days.

Corneal damage was evaluated and graded in a masked fashion using a scoring system described previously by Keadle et al. [17]. Briefly, on the designated days after UVB irradiation, stromal opacification was rated on a scale of 0–4 where 0 indicates clear stroma, 1 indicates mild stromal opacification, 2 indicates moderate opacity with discernible iris features, 3 indicates dense opacity with loss of defined iris detail except pupil margins, and 4 indicates total opacity with no posterior view.

### Tissue preparation and nuclear protein extraction

Four, seven, ten, fourteen, and twenty-one days after UVB irradiation, the mice were humanely sacrificed by cervical dislocation and whole eyes were removed immediately. Under an operating microscope, corneas were quickly dissected without any limbal or iris tissue for analysis of NF-κB and TNF-α. Excised corneas were frozen in liquid nitrogen and stored at −80 °C until analysis. Corneas obtained from mock-infected mice at day 14 were included as controls. For preparation of nuclear extracts, six corneas having reactivated lesions (confirmed by slit-lamp biomicroscopy and virus-positive eye-swab data) were collected and minced at designated time points. The nuclear extracts of two cornea tissues were pooled and prepared by hypotonic lysis followed by a high-salt extraction. Frozen corneas were rinsed with ice-cold buffer A, which contained 10 mM HEPES (pH 7.9), 10 mM KCl, 2 mM MgCl_2_, 0.1 mM EDTA, 1.0 mM dithiothreitol (DTT), and 0.5 mM phenylmethylsulfonyl fluoride (PMSF). All chemicals were from Sigma Chemical Co. (St Louis, MO). The homogenate was incubated on ice for 20 min after 50 µl of 10% Nonidet P-40 was added. The mixture was vortexed for 30 s and centrifuged (5000x g) for 1 min at 4 °C. Supernatants were harvested and stored in small aliquots at −80 °C for analysis of TNF-α. The crude nuclear pellets were re-suspended in 200 µl of ice-cold buffer B containing 20 mM HEPES (pH 7.9), 420 mM NaCl, 0.1 mM EDTA, 1.5 mM MgCl_2_,1 mM DTT, 0.5 mM PMSF, and 25% (v/v) glycerol and were incubated on ice for 30 min with intermittent mixing. The lysates were then centrifuged at 12000x g for 20 min at 4 °C, and the supernatants containing the nuclear proteins were stored at −80 °C for NF-κB analysis. Protein concentrations were determined using a Bradford protein assay kit (Bio-Rad, Hercules, CA).

### Electrophoretic mobility shift assay

The NF-κB DNA–binding activity was measured by electrophoretic mobility shift assay (EMSA) according to the manufacturer’s instructions (Gel Shift Assay Systems; Promega, Madison, WI). The NF-κB oligonucleotide was end-labeled with [γ-^32^P] ATP (Free Biotech, Beijing, China) using T4-polynucleotide kinase. Binding reactions were performed by adding 10 µg of nuclear extracts to 7 µl of binding buffer containing 10 mM Tris-HCl (pH 7.5), 1 mM MgCl_2_, 0.5 mM EDTA, 0.5 mM DTT, 50 mM NaCl, 4% glycerol, 0.05 g/l polydeoxyinosinic deoxycytidylic acid, and 1 µl of [γ-^32^P]ATP-labeled oligonucleotide probes. The binding specificity of the DNA/protein binding was determined by competitive reactions in which a 100 fold molar excess of unlabeled NF-κB oligonucleotide was added to the binding reaction 10 min before the addition of the biotin probe. Mixed samples were incubated at room temperature (25 °C) for 30 min and fractionated by electrophoresis on a 4% non-denaturing polyacrylamide gel in a TBE (Tris-borate EDTA) buffer that was pre-electrophoresed for 1 h at 100 V. After electrophoresis, gels were dried and autoradiographed to detect NF-κB binding activity.

### Enzyme-linked immunosorbent assay analysis of TNF-α

TNF-α protein was assayed using a commercial enzyme-linked immunosorbent assay (ELISA) kit (Biosource International Inc., Camarillo, CA) following the manufacturer’s instruction with the sensitivity being 5 pg/ml.

### Histopathological evaluation and immunocytochemistry

Enucleated eyes 14 days after irradiation were placed in 10% buffered neutral formalin and embedded in paraffin. The 5 μm sagittal sections were then cut, and some of the sections were stained with Hematoxylin and eosin (H&E) for a histological examination under a microscope. For immunocytochemical analysis, sections cut from the same paraffin blocks were deparaffinized and rehydrated. Slides were boiled in a target retrieval solution buffer (Dako, Kyoto, Japan) in a microwave for 4 min and then placed at room temperature (25 °C) for 20 min. Endogenous peroxidase activity was blocked by addition of 0.3% hydrogen peroxidase in methanol, and the sections were treated with normal goat serum and incubated overnight with a monoclonal antibody against the p65 subunit of activated NF-κB (1:100; Santa Cruz Biotechnology, Santa Cruz, CA) at 4 °C. Thereafter, a biotinylated secondary antibody against mouse IgG and an avidin-biotinylated peroxidase complex (Santa Cruz Biotechnology) were used with 3,3′-diaminobenzidine as a peroxidase substrate. Sections were counterstained with hematoxylin. Negative controls were performed by omitting the primary antibody.

### Statistical analysis

χ^2^ analysis was used to compare the reactivation rates. The Wilcoxon-Mann–Whitney rank sum test was used for comparison of corneal opacity scores at every time point. The data from EMSA and ELISA were expressed as mean±standard error (SE). Differences between groups were determined by Student’s *t*-test. A p<0.05 was considered statistically significant.

## Results

### Effects of LOR on the incidence of recurrent virus shedding and reactivation rate

As shown in Table 1, the daily LOR treatment was associated with the reduction in the positive virus shedding following UVB-stimulated ocular HSV recurrence. The positive virus shedding in LOR-treated mice was significantly lower than that in saline control mice, especially on days 3, 4, and 6. There was no positive viral shedding in either the mock infected control or LOR control mice. A significant reduction in the overall reactivation rate was observed in the mock immunized mice (33/76, 43.4%) compared with the saline control group (53/76, 69.7%; Table 1; p=0.001).

**Table 1:** Numbers of mice in the different treatment groups with viral shedding and stromal disease.

**Treatment group** ** **	**Positive virus shedding^a^ (%)**								**Stromal disease^b^ (%)**			**Reactivation rate^c^**
	**D1**	**D2**	**D3**	**D4**	**D5**	**D6**	**D7**	**Total**	**D4**	**D7**	**Total**	** **
Saline treatment	0/76 (0)	12/76 (15.8)	29/76 (38.2)	46/76 (60.5)	22/70 (31.4)	10/70 (14.3)	2/70 (2.8)	58/76 (76.3)	37/76 (48.7)	47/70 (67.1)	61/76 (80.2)	53/76 (69.7)
Lornoxicam treatment	0/76 (0)	8/76 (10.5)	17/76* (22.4)	28/76* (36.8)	13/70 (18.6)	2/70* (2.8)	0/70 (0)	37/76* (48.7)	17/76* (22.4)	27/70* (38.6)	37/76* (48.7)	33/76* (43.4)

Eyes of mice with latent ocular HSV infection were UVB irradiated (day 0) and swabbed to detect recurrent viral shedding from days 0 to 7. The eyes were observed under a slit-lamp biomicroscope in a masked fashion. In the table, "^a^" indicates the number of eyes shedding virus versus total number of eyes irradiated with UVB and "^b^" indicates the incidence of stromal opacity in each group. "^c^" indicates recurrent disease was defined as stromal opacification for more than two consecutive days and virus shedding in tears on any day from day 1 to day 7 post-UVB exposure with day 0 swabs serving as the control. D=days post UVB irradiation. An asterisk indicates that there was a significant difference when compared with the saline-treated group at the corresponding time points (range p<0.001–0.080).

### Effects of LOR on the tissue lesions

**Figure 1:**
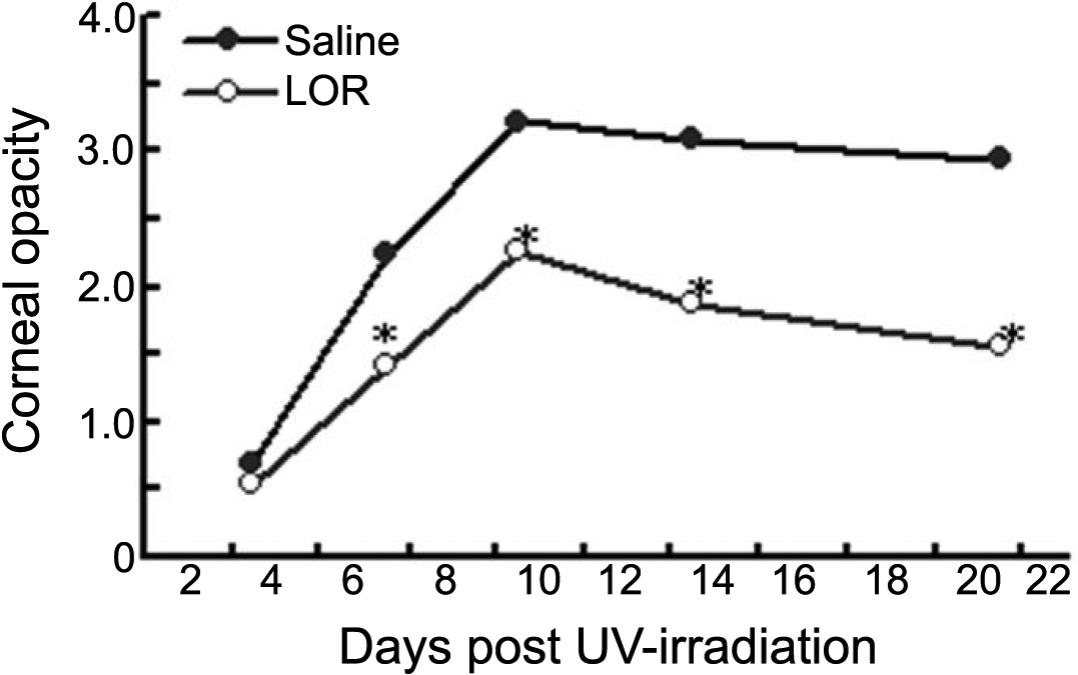
Lornoxicam treatment decreases the severity of recurrent herpetic stromal keratitis. Latently infected mice were consecutively treated with 0.4 mg/kg of lornoxicam (LOR) or an equal volume of saline on one day before UVB exposure and repeated on the following six days post-UVB exposure. The mean score of corneal opacity in LOR-treated mice (HSK+LOR) was significantly lower than that in the saline control mice (HSK), reaching significance on days 7 to day 21 (p=0.09). The asterisk indicates that p<0.001 when compared to the HSK group at the corresponding time points.

As shown in Table 1, on day 7 after UVB irradiation, the development of stromal disease was detected in 67.1% (47/70) of the mice in the saline-treated group but only 38.6% (27/70) in the LOR-treated group. This difference was statistically significant (p=0.001). Similar results were obtained on day 4 after UVB irradiation (Table 1). Moreover, significant corneal opacity was observed in reactivated eyes of mice treated with saline. In contrast, mice receiving LOR demonstrated a significant reduction in clinical scores compared to the saline-treated group (Figure 1). Although 82.86% of the saline-treated eyes developed clinically evident lesions (score 3 or greater) at day 14 after recurrence, only 13.33% of eyes treated with LOR developed such lesions. Mock-infected mice after UVB irradiation developed mild and transient opacity, which disappeared within four days (data not shown). Taken together, these results indicate that LOR reduced the development of keratitis via inhibition of inflammation.

### Effect of LOR on NF-κB activation in the cornea

**Figure 2:**
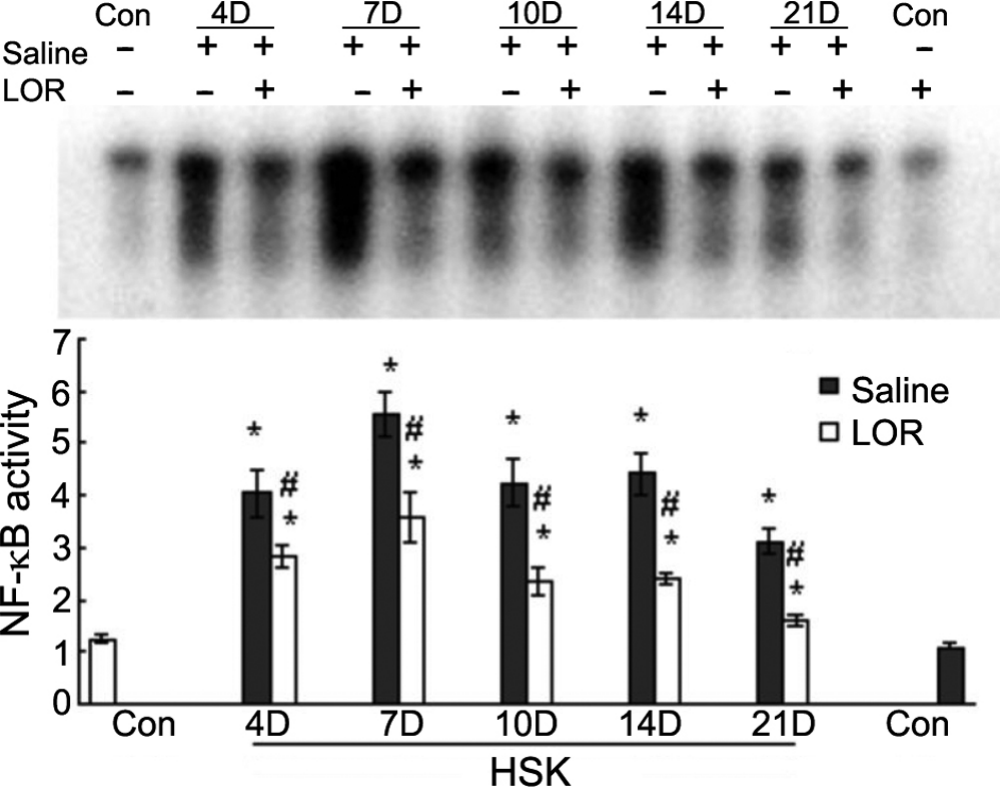
Electrophoretic mobility shift assessment of the DNA-binding activity of NF-κB in the cornea of different groups. **A**: Nuclear extracts were probed for NF-κB binding activity. Lane 1: mock-infected group (Control); lanes 2, 4, 6, 8, and10: Reactivated, saline-treated group (HSK) on days 4, 7, 10, 14, and 21, respectively; lanes 3, 5, 7, 9, and 11: Reactivated, LOR-treated group (HSK+LOR) on days 4, 7, 10, 14, and 21, respectively; lane 12: mock-infected group treated with lornoxicam (LOR). The data are representative of three independent experiments. **B**: Quantitative analysis is displayed of NF-κB activity by EMSA at days 4, 7, 10, 14, and 21 after irradiation in corneas of ICR mice treated with either saline or LOR. The bands were quantified using image analysis software (*Bandleader* 3.0 software, *Magnitec *Ltd. Tel Aviv, Israel). The relative intensity was determined by comparison with that of the background. The data are presented as mean±standard error of results from three independent experiments. EMSA shows a markedly upregulated activity of NF-κB in the HSK group and HSK+LOR group compared to levels in the control group, and this upregulation was dramatically suppressed by LOR treatment at each indicated time point. The asterisk indicates that p<0.05 when compared to the control group. The hash mark indicates that p<0.05 for the HSK+LOR group when compared to the HSK group at the corresponding time points.

To evaluate whether LOR diminished the activity of NF-κB, we performed EMSA to determine the levels of nuclear NF-κB DNA–binding activity in the cornea after recurrence. As shown in the EMSA blot, there was a constitutive low expression in the mock-infected group but prominent high intensity in the saline-treated, infected group (Figure 2). HSV-1 recurrence resulted in a significant upregulation of NF-κB activity, shown as a maximal fivefold induction of NF-κB compared with the mock-infected group. Moreover, an obvious decrease in NF-κB activity was found in the LOR-treated group compared to the saline-treated group. Since no differences were observed in NF-κB activity between the mock infected saline-treated group and the LOR alone group, it seemed that LOR alone did not have any significant effect on the activity of NF-κB.

### Effect of LOR on TNF-α expression in the cornea

**Figure 3:**
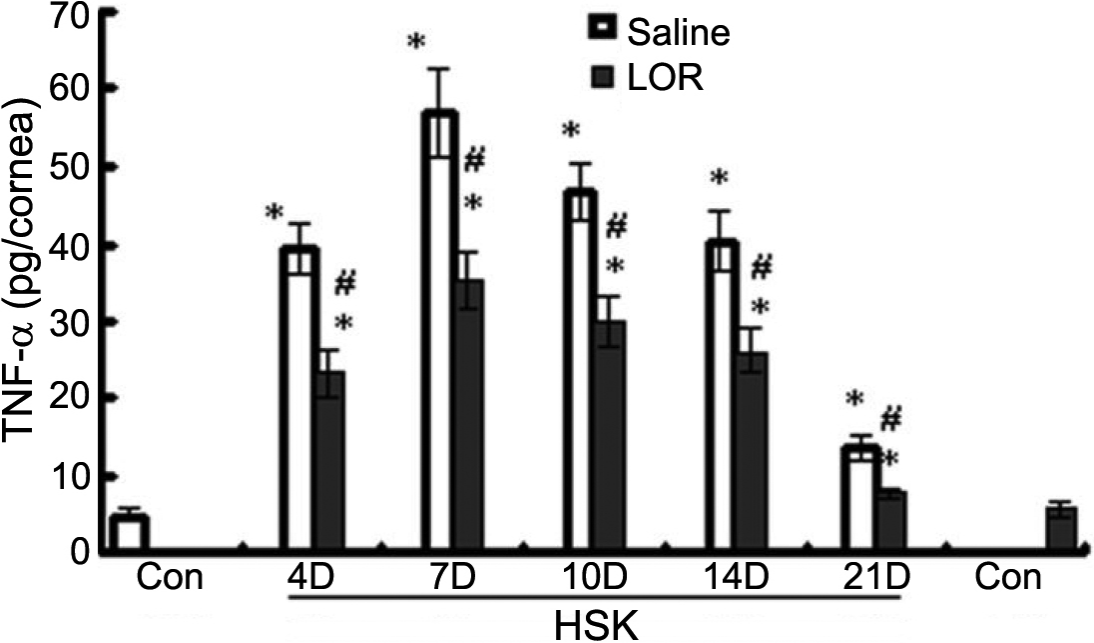
TNF-α expression in corneas of different groups determined by ELISA. The data are presented as the mean±standard error of results from three independent experiments. ELISA showed a markedly upregulated activity of NF-κB in infected corneas of saline-treated mice (HSK) and LOR-treated mice (HSK+LOR) when compared to levels in the mock-infected cornea (Control). LOR treatment significantly inhibited TNF-α expression in the cornea at each indicated time point. The asterisk indicates that p<0.05 when compared to the control group, and the hash mark denotes that p<0.05 for the HSK+LOR group when compared to the HSK group at the same time point.

As shown in Figure 3, the kinetics and pattern of elevated TNF-α expression were similar to that of HSV-induced NF-κB activity. The TNF-α expression in all infected groups were significantly higher than that in the mock-infected control, showing a maximal 11.6 fold increase in comparison with the mock-infected control at day 7. In contrast, the production of TNF-α was markedly suppressed by LOR treatment before recurrence. Cornea from the mock-infected mice and from the LOR alone mice showed minimal basal levels of TNF-α expression (p=0.128).

### Effect of LOR on keratitis by histology

**Figure 4:**
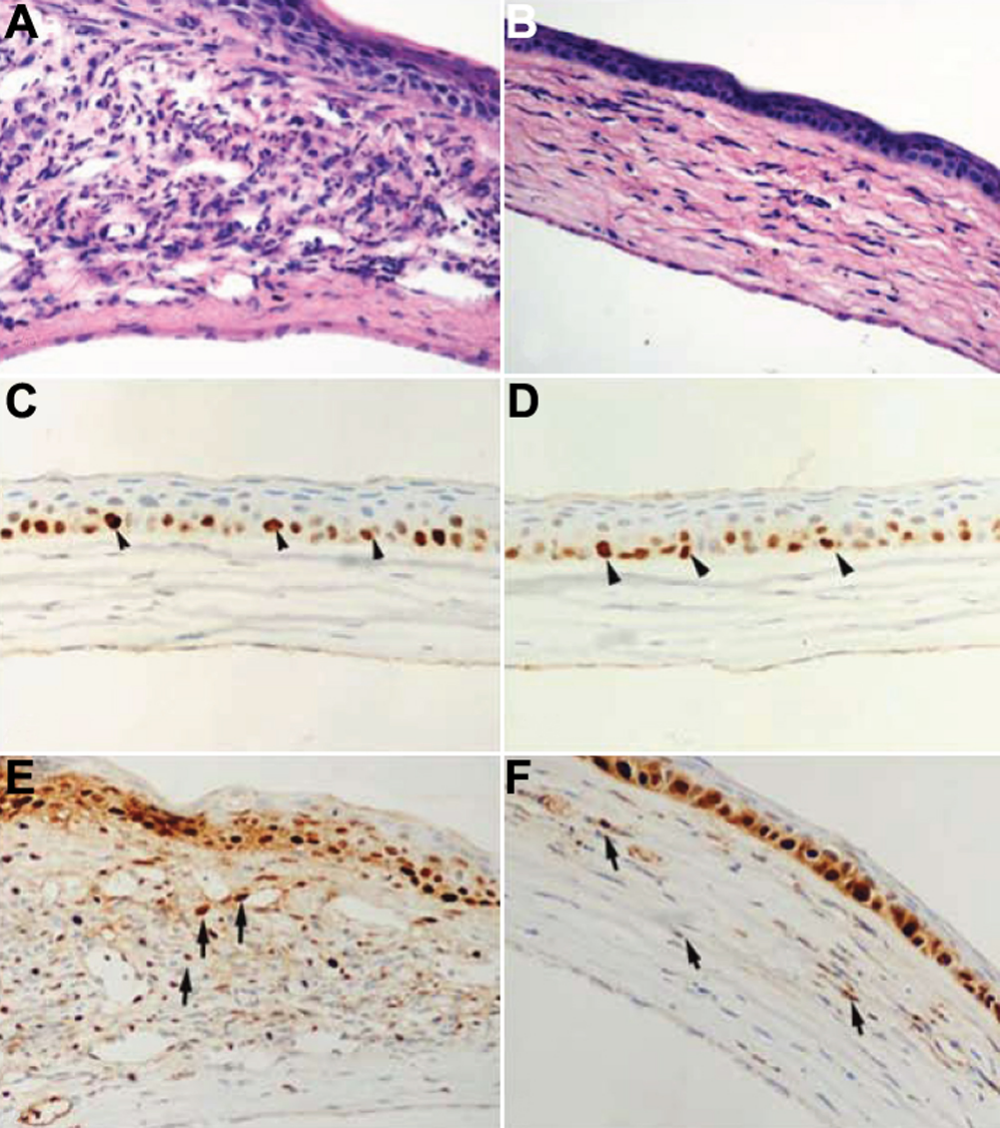
Influence of LOR on the histopathological and immunohistochemical studies in mouse corneas 14 days after UV irradiation. The typical histologic findings of cornea stained with hematoxylin and eosin (**A**, **B**) are shown. **A**: The cornea in the saline-treated group shows marked inflammation, obvious edema, profound neovascularization, and significant hypercellularity in the stroma. **B**: The cornea in the LOR-treated group exhibits only scattered inflammatory cells, mild stromal swelling, and less neovascularization. Corneal tissues (**C**-**F**) were analyzed by immunohistochemistry to determine the expression of NF-κB. Immunohistochemical staining with an antibody against activated NF-κB was performed to detect the expression of NF-κB. Sections incubated without a primary antibody served as negative controls. All tissue sections were counterstained with hematoxylin. These samples were representative of all corneas examined. Brown staining indicates activated NF-κB. **C**,**D**: The cornea in the mock-infected group and the cornea in LOR alone group show that NF-κB activity is only observed very faintly in the base cells of epithelium (arrowheads). **E**: Recurrence induced wide spread positive staining of NF-κB, which was most robust in the stroma (arrows) of the saline-treated group. **F**: Scant immunoreactivity of NF-κB was observed in the stroma of the LOR-treated group (arrows). Original magnifications, 400X.

As shown in Figure 4, the corneas experiencing recurrent HSK showed significant extracellular edema, pronounced infiltrate, and congestive blood vessels 14 days after irradiation whereas LOR treatment ameliorated the recurrent HSK lesions. A significantly decreased number of inflammatory cells and less neovascularization were observed in the LOR-treated group (Figure 4A,B).

### Effect of LOR on activated NF-κB immunoreactivity in the cornea

Immunohistochemical staining was performed with an antibody against the activated p65 subunit of NF-κB to evaluate the distribution of NF-κB in the cornea. A similar expression pattern of NF-κB in corneas from the control (Figure 4C) and LOR alone groups (Figure 4D) was observed, showing only a faint stain for p65 in the nuclei of the basal corneal epithelial cells. No keratocytes with immunoreactivity for activated p65 were detected in the stroma. In contrast, intense and diffuse positive staining for p65 was demonstrated in the basal corneal epithelial cells and throughout the stroma in the reactivated, saline-treated group (Figure 4E). Meanwhile, immunoreactivity of p65 was scant and reduced in the epithelium and stroma in the LOR-treated cornea as compared with the saline-treated cornea (Figure 4F). LOR effectively blocked the nuclear translocation of NF-κB. Immunoreactivity was not detectable when the primary antibody was omitted (data not shown), indicating that the reaction was specific.

## Discussion

The pathogenesis of recurrent HSK has become an area of intense research. It has been widely realized that the progressive visual impairment induced by reactivated HSV-1 infection is largely due to the immune-mediated inflammatory process rather than the cytopathic effects. Therefore, it has been of interest to treat recurrent HSK with anti-inflammatory drugs.

Our results have shown that LOR, a COX inhibitor, could not only provide protection against HSK recurrences but also exert a beneficial effect on the prognosis of recurrent HSK in a mouse model. Moreover, to the best of our knowledge, this was the first study demonstrating that LOR exerted its effects through the inhibition of NF-κB and subsequent decrease in expression of cytokines, reducing inflammatory response.

At the cellular level, LOR likely prevents recurrence and inhibits stromal opacity directly by reducing the expression of cytokines. Consistent with previous reports [7], we observed a significant upregulation of TNF-α level in mice with UVB-induced HSV reactivation [16,17]. TNF-α, whose expression is regulated by NF-kB activation, is among the first to be produced in chronic inflammation and tissue damage during the development of HSK [7,8,18]. It enhances synthesis of inflammatory mediators and contributes to the recruitment of inflammatory cells. In this study, LOR inhibited the expression of TNF-α as well as the development of corneal inflammation.

Given the pivotal role NF-κB plays in the immune system and inflammatory diseases, we investigated its activation during recurrent HSK in mice. Consistent with several reports addressing the role of NF-κB activation in corneal neovascularization and lipopolysaccharide-induced keratitis [19,20], we clearly demonstrate that there is a significant and persistent upregulation of activated NF-κB in the cornea during HSK recurrence. The NF-κB p65 immunoreactivity was associated with corneal inflammation leading to neovascularization and opacity. The ameliorated clinical lesion of HSK after LOR treatment was accompanied by a dramatic reduction in recurrence-induced activation of NF-κB. At the molecular level, the increased expression of TNF-α is due to the activation of the transcriptional factor, NF-κB [7,8]. On the other hand, TNF-α can act as a potent inducer of NF-κB activation [21]. Then sustained NF-κB activation and elevated expression of TNF-α trigger the perpetuated inflammatory reactions. In the present study, we have demonstrated that LOR treatment blocked signaling through NF-κB, dampened the pro-inflammatory response to HSV-1, and thereby reduced corneal opacity. Previous reports have shown that NF-κB can function as an upstream regulator of *COX-2*, controlling its transcription [22,23]. COX inhibitors can exert their anti-inflammatory activities by blocking the NF-κB signaling pathways via one or several steps in the activation cascade [24-27]. Moreover, the non-steroidal anti-inflammatory drugs (NSAIDs) may exert their effects via the NF-κB pathway, independent of COX inhibition [28]. The concrete mechanism by which LOR inhibits the NF-κB activation has yet to be elucidated.

Previous reports have unveiled that HSV-1 interferes with cellular signal transduction and transcription factor activity, especially the activation of NF-κB, thus promoting viral replication and expression of pro-inflammatory proteins. Accordingly, HSV-1 infection can act as a stimulant for NF-κB expression. Moreover, this persistent activation of NF-κB is one of the immune evasion tactics that HSV-1 has acquired to limit the efficacy of innate or acquired immunity [9-12]. As such, the suppressed viral replication may be mediated through the inhibition of NF-κB activity. In support of this concept, glucocorticoid, resveratrol, and tetranortriterpenoid treatment have been shown to effectively inhibit the activation of NF-κB and thereby effectively suppress HSV-1 infection [29-32]. UVB irradiation functions as an inducer for viral reactivation in the recurrent HSK model. We speculate that UV irradiation induces the elevated NF-κB activation and subsequent mediator production, leading to herpes viral reactivation. Although we have shown in a previous study the therapeutic effect of lornoxicam on corneal damage after high intensity UVB irradiation via inhibition of NF-kB activation, the concrete mediators and detailed mechanisms between NF-κB activation and viral reactivation need further investigation [33]. Since patients undergoing excimer laser keratectomy or penetrating keratoplasty are at high risk for developing herpes viral infection or reactivation [34-37], further studies will address the potential efficacy of prophylactic LOR treatment in those patients.

Of note, since NF-κB is essential in maintaining normal host defense and broadly involved in multiple cell regulation [29,38], precautions should be taken when considering the targeting NF-kB as a novel therapeutic approach. In the current study, valuable information is provided by the LOR alone groups in which the NF-κB signaling pathway was selectively unaffected under a similar physiologic response after LOR treatment. Although we did not observe any abnormalities in mice that were systemically administered LOR until the end of the experiment, it is uncertain if undesirable effects may occur after long-term usage.

In conclusion, based on previous reports showing antiviral activity of COX inhibitors against ocular infection with HSV-1 [4-6], we extended those previous findings by exploring the intrinsic mechanism underlying the protective activity of LOR. The results reported herein may shed new light on the development of novel therapeutic and prophylactic approaches to treating recurrent HSK.
